# Loss of a single allele for Ku80 leads to progenitor dysfunction and accelerated aging in skeletal muscle

**DOI:** 10.1002/emmm.201101075

**Published:** 2012-08-23

**Authors:** Nathalie Didier, Christophe Hourdé, Helge Amthor, Giovanna Marazzi, David Sassoon

**Affiliations:** 1 Myology Group, UMR S787 INSERM, Université Pierre et Marie Curie Paris VIPitié-Salpétrière, Paris Cedex, France; 2 Institut de Myologie, Université Pierre et Marie Curie Paris VI, Unité Mixte de Recherche UPMC-AIM UM 76INSERM U 974, CNRS UMR 7215, Paris, France

**Keywords:** aging, Ku80, skeletal muscle, stem cells, telomere

## Abstract

Muscle wasting is a major cause of morbidity in the elderly. Ku80 is required for DNA double strand repair and is implicated in telomere maintenance. Complete loss-of-function leads to reduced post-natal growth and severe progeria in mice. We examined the role of Ku80 in age-related skeletal muscle atrophy. While complete loss of Ku80 leads to pronounced aging in muscle as expected, accompanied by accumulation of DNA damage, loss of a single allele is sufficient to accelerate aging in skeletal muscle although post-natal growth is normal. Ku80 heterozygous muscle shows no DNA damage accumulation but undergoes premature telomere shortening that alters stem cell self-renewal through stress response pathways including p53. These data reveal an unexpected requirement for both Ku80 alleles for optimal progenitor function and prevention of early onset aging in muscle, as well as providing a useful model for therapeutic approaches.

## INTRODUCTION

Skeletal muscle aging is characterized by myofibre atrophy (Brooks & Faulkner, 1994; Einsiedel & Luff, 1992) coupled with a decline in regenerative capacity (Grounds, 1998). Reversible age-related changes have been identified in the stem cell niche such as altered notch signalling (Conboy et al, 2003, 2005), however, irreversible age-related accumulation of DNA damage and telomere shortening leading to cell stress responses are also proposed to contribute to stem cell dysfunction and subsequent loss of regenerative capacity in several tissues (Blasco et al, 1997; Flores et al, 2005; Nagley et al, 1992; Rossi et al, 2007; Sacco et al, 2010; Vijg, 2000). p53 activation results from DNA damage and telomere shortening leading to cell cycle arrest, death or senescence (Chin et al, 1999; Itahana et al, 2001; Vousden, 2000). p53 also regulates aging and longevity (Pinkston et al, 2006; Vaziri et al, 2001). Mouse mutants for DNA repair enzymes exhibit chronic p53 activation coupled with premature aging and are partially rescued in a p53 mutant background (Cao et al, 2003; Varela et al, 2005; Vogel et al, 1999). p53 regulates stem cell self-renewal in a variety of tissues including skeletal muscle (Cicalese et al, 2009; Dumble et al, 2007; Lin et al, 2005; Liu et al, 2009; Meletis et al, 2006; TeKippe et al, 2003; Tyner et al, 2002). We showed previously that p53 and a p53 downstream effector, PW1, are expressed in myogenic progenitors and are required for TNF-mediated inhibition of muscle differentiation (Coletti et al, 2002; Relaix et al, 1998, 2000; Schwarzkopf et al, 2006). p53 mutant mice are resistant to muscle wasting induced by tumour load (Schwarzkopf et al, 2006). Although p53 is not essential for skeletal muscle development nor regeneration, p53 deletion alters the stem cell number in adult tissues including muscle (Donehower et al, 1992; Schwarzkopf et al, 2006; White et al, 2002). Whereas chronic cell stress responses including p53 activation promote aging, the IGF-1/Akt pathway promotes longevity. In response to cell stress, p53 induces genes that negatively regulate the IGF-1/Akt pathway (Feng et al, 2007). The Foxo family of Forkhead transcription factors are major downstream effectors of IGF-1/Akt pathway, and regulate cell cycle and DNA repair (Barthel et al, 2005). One Foxo family member, Foxo1 (FKHR), stimulates myogenesis (Bois & Grosveld, 2003) and regulates fibre type as well as muscle mass (Kamei et al, 2004).

Given the link between p53 and aging, we investigated the role of p53 in age-associated muscle atrophy using the Ku80 mutant mouse model. Ku80 null mutants exhibit premature age-specific changes including osteopoenia, atrophic skin and shortened life span (Vogel et al, 1999). Ku80 is part of the Ku heterodimer complex that acts with the DNA dependent protein kinase (DNA-PK) catalytic subunit in the non-homologous end-joining pathway (NHEJ), a key mechanism for repairing DNA double-strand breaks in mammals providing genomic stability. In addition to a role in DNA repair, Ku80 localizes to telomeres suggesting a role in the maintenance of telomere stability (D'Adda di Fagagna et al, 2001). We show that Ku80 null and heterozygous skeletal muscles exhibit early onset aging. Whereas the Ku80 null mice display a catastrophic aging phenotype, our data reveal an unexpected phenotype in Ku80 heterozygous muscle that closely resembles accelerated but physiological aging. We propose that telomere shortening rather than accumulated DNA damage is responsible for age-associated muscle stem cell dysfunction and consequent loss of regenerative capacity. We show that p53 and IGF-I/Akt pathways intersect, leading to cell cycle checkpoint impairment and decreased stem cell self-renewal. The initial normal growth of Ku80 heterozygous mice followed by an early onset aging phenotype provides a model for further investigation into sarcopenia and therapeutic approaches.

## RESULTS

### Loss of a single allele for Ku80 leads to premature muscle aging

**Figure 1:**
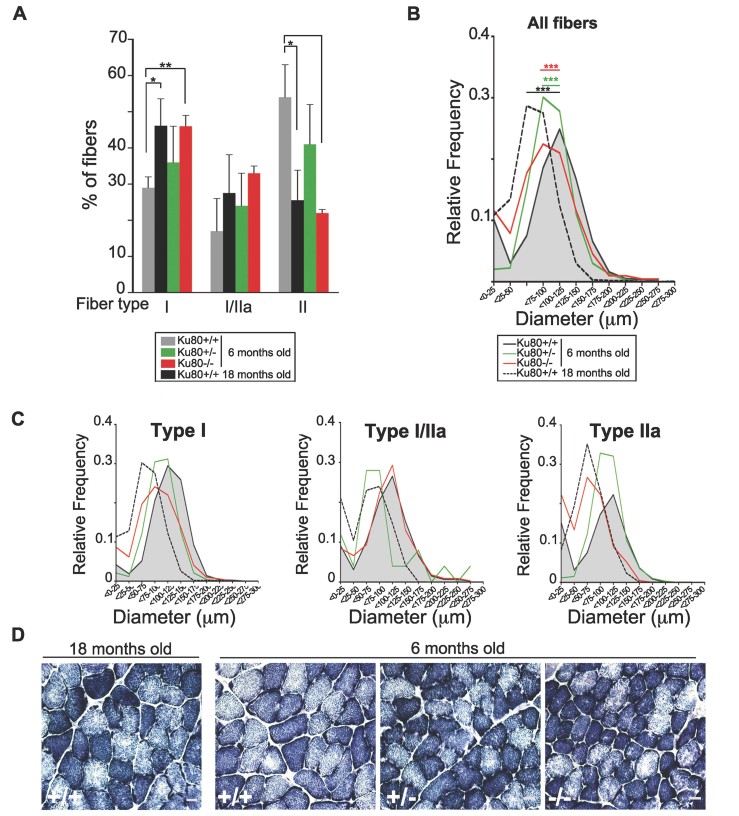
Loss of Ku80 leads to premature muscle aging Proportions of MHC isoforms expressed in 6-month-old Ku80^+/+^, Ku80^+/−^ and Ku80^−/−^ and 18-month-old Ku80^+/+^ *Soleus*. 18-month-old Ku80^+/+^ and Ku80^−/−^ *Soleus* exhibits significant changes in slow (*p = 0.03 and **p = 0.007) and fast fibres proportions (*p = 0.03) compared to Ku80^+/+^.Fibre size distribution in 6-month-old Ku80^+/−^ (green), Ku80^−/−^ (red) and Ku80^+/+^ (grey) and 18-month-old Ku80^+/+^ (dash) *Soleus* (***p < 0.0001, 6-month-old Ku80^+/+^ *vs*. 18-month-old Ku80^+/+^, Ku80^+/−^ and Ku80^−/−^).Fibre size distribution correlated to fibre type. 18-month-old Ku80^+/+^ *Soleus* exhibited a marked reduction of type I and type IIa fibre size compared to 6-month-old Ku80^+/+^ (***p < 0.0001). Ku80^+/−^ *Soleus* exhibited a reduced size of type I (*p = 0.04) and IIa (***p < 0.0001) fibres compared to Ku80^+/+^. Ku80^−/−^ *Soleus* exhibited a marked reduction of type I (***p < 0.0001) and type IIa (*p = 0.02) fibre size compared to Ku80^+/+^.Representative photomicrographs of *Soleus* cross-section from 6-month-old Ku80^+/+^, Ku80^+/−^ and Ku80^−/−^ mice, and 18-month-old Ku80^+/+^ mice stained for NADH-TR diaphorase reaction. 18-month-old Ku80^+/+^, Ku80^+/−^ and Ku80^−/−^ muscles exhibit an increased proportion of oxidative fibres (dark blue) as compared to 6-month-old Ku80^+/+^ muscle. Scale bar = 20 µm.

Complete loss of Ku80 leads to premature aging whereas no phenotype has been reported for the heterozygous mice (Lim et al, 2000; Vogel et al, 1999). We anticipated a similar phenotype for skeletal muscle. Decreased fibre size and a switch in myosin heavy chain (MHC) isoforms from ‘fast’ to ‘slow’ are hallmarks of skeletal muscle aging (Brooks & Faulkner, 1994; Doran et al, 2009; Einsiedel & Luff, 1992; Larsson et al, 1993; Schiaffino & Reggiani, 1994). Histological analyses of proportions of slow (type I), fast (type IIa) and mixed (type I/IIa) fibres were correlated to fibre size on *Soleus* muscle from 6-month-old Ku80 wildtype, heterozygous and null mice and 18-month-old (aged) Ku80 wildtype mice. We observed that Ku80 null *Soleus* muscle as well as *Soleus* from aged Ku80 wildtype mice exhibited an increase in the proportion of slow fibres associated with a decrease in the proportion of fast fibres as compared to wildtype (Fig 1A). As expected, aged Ku80 wildtype *Soleus* exhibit a decreased size in all fibre type as compared to 6-month-old Ku80 wildtype *Soleus* (Fig 1B and C). As reported previously, Ku80 null mice are smaller than wildtype (Nussenzweig et al, 1996), and the Ku80 null *Soleus* exhibited greatly reduced fibre sizes as compared to Ku80 heterozygous and wildtype muscles (Fig 1B). In contrast, Ku80 heterozygous mice do not exhibit a significant difference in body size [(Nussenzweig et al, 1996) and Supporting Information Fig S1A]. However, the Ku80 heterozygous *Soleu*s displayed a modest size reduction in all fibre types as compared to wildtype (Fig 1B and C), thus changes in fibre size are not linked to body size. Consistent with the changes observed in fibre types, we observed an increase in mitochondrial density reflecting a loss of fast fibres and an increase in smaller slow fibres (Fig 1D). An additional feature of aging is a reduction in cell number (Nijnik et al, 2007; Tyner et al, 2002). While we detected no significant differences in the overall number of muscle fibres in all cases (Supporting Information Fig S1B), we found that the total nuclei number was decreased in both the sublaminal and interstitial compartments of neonatal Ku80 null muscle as compared to heterozygous and wildtype muscle (Supporting Information Fig S1C). By 15 days after birth, we observed a decline in sublaminal nuclei in all genotypes examined. However, the number of interstitial nuclei markedly decreased in Ku80 heterozygote and null muscles, but remained less affected in wildtype muscle (Supporting Information Fig S1C). These observations reveal an unanticipated haplotype insufficiency in Ku80 heterozygous mice.

### Regenerative capacity is impaired with age and in Ku80 heterozygous and null muscles

**Figure 2:**
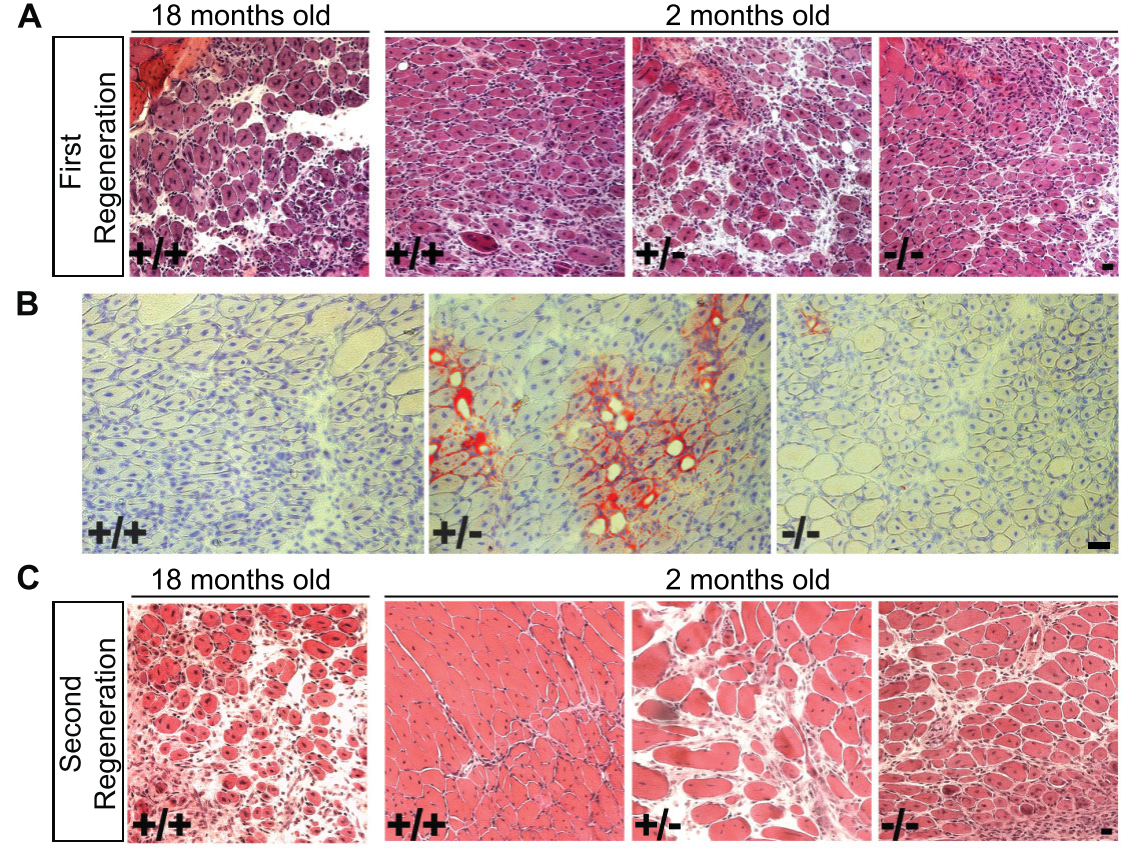
Regenerative capacity is impaired with age and prematurely impaired in Ku80 heterozygous and null muscles *TA* cross-sections 7 days after injury stained with hematoxylin/eosin. Regenerative capacity decreases with age in Ku80^+/+^ muscles. Ku80^−/−^ muscle regeneration is more efficient than in Ku80^+/+^ muscle, while Ku80^+/−^ muscle regeneration is impaired. Scale bar = 20 µm.Representative photomicrographs of 2 months *TA* cross-sections 7 days after injury stained with Oil Red O and hematoxylin. Adipocytes accumulate between regenerating fibres in Ku80^+/−^ muscles as compared to Ku80^+/+^ and Ku80^−/−^ muscles. Scale bar = 20 µm.Cross sections from serially injured TA 7 days after the second injury. Regeneration is less efficient in 18-month-old Ku80^+/+^ and in 2-month-old Ku80^+/−^ and Ku80^−/−^ muscles following serial injury as compared to 2-month-old Ku80^+/+^.

We induced focal injuries on the *Tibialis Anterior* (*TA*) to test regenerative capacities of 2-month-old Ku80 wildtype, heterozygous and null mice and analysed 7 days following injury, corresponding to a stage when damaged tissue is cleared and nascent fibres are formed (Charge & Rudnicki, 2004). We compared our results with 18-month-old wildtype injured muscles. As reported by others (Brack et al, 2005; Conboy et al, 2003; Grounds, 1998), we observed a decrease in the regenerative capacity of 18-month-old wildtype muscle as compared to 2-month-old wildtype muscle reflected by larger interstitial spaces and increased number of infiltrating cells (Fig 2A). In contrast, we found that Ku80 null muscle regenerated more rapidly than 2-month-old wildtype muscles (Fig 2A) whereas Ku80 heterozygous muscle showed poor regeneration and accumulation of adipocytes (Fig 2A and B). These data reveal a paradoxical accelerated regeneration in Ku80 null muscle and a decline in regenerative capacity in heterozygous muscle. We next tested regenerative potential following two rounds of injury as an indirect measure of stem cell self-renewal. The TA of adult Ku80 wildtype (2- and 18-month-old), heterozygous and null mice were focally injured twice with an interval of 15 days and analysed 1 week after the second injury. Under these conditions, we observed severely reduced regeneration in 18-month-old wildtype muscles compared to 2-month-old wildtype. Ku80 heterozygous and null muscles displayed a reduced regeneration as compared to wildtype (Fig 2C). Furthermore, we observed more fibrotic tissue in Ku80 null muscle (Supporting Information Fig S2).

### Ku80 null and heterozygous progenitors display altered self-renewal properties

**Figure 3:**
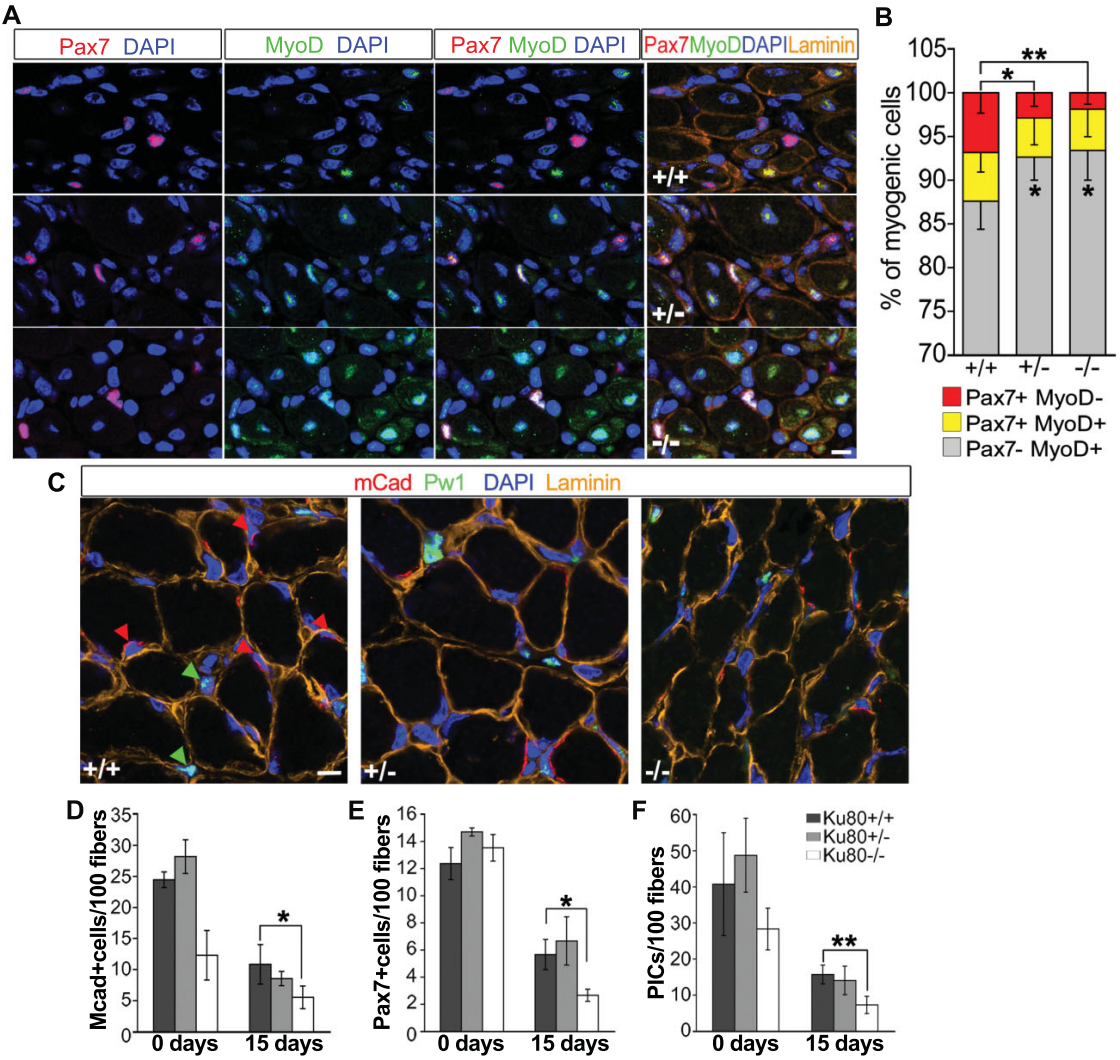
Ku80 heterozygous and null progenitors display altered self-renewal **A.** Representative cross-sections of serial injured muscles immunostained for Pax7 (red), MyoD (green), Laminin (orange), and DAPI (blue) to visualize the nuclei. Scale bar = 10 µm.**B.** Histogram showing proportions of myogenic cells Pax7^+^/MyoD^−^ (self-renewing), Pax7^+^/MyoD^+^ (proliferating), or Pax7^−^/MyoD^+^ (committed) quantified from sections stained as shown in (**A**). The proportion of self-renewing cells is decreased in Ku80^+/−^ and Ku80^−/−^ compared to Ku80^+/+^, whereas the committed myoblasts show a concomitant increase (*p = 0.011, **p = 0.006).**C.** Cross-sections of 15 days old hindlimb muscles immunostained for M-cadherin (red), PW1 (green) and Laminin (orange). Nuclei were visualized by DAPI (blue). Red arrows: M-cadherin^+^ satellite cells. Green arrows: PICs. Scale bar = 10 µm.**D-F.** Histograms showing quantification of M-cadherin (**D**) and Pax7 positive (**E**) satellite cells and PICs (**F**) from cross-sections stained as shown in (**C**). Between birth and 15 days, the number of M-cadherin^+^ and Pax7^+^ satellite cells and PICs is decreased, however this decrease is more pronounced in 15 days old Ku80^−/−^ muscle compared to Ku80^+/+^ (*p = 0.02, *p = 0.01 and **p = 0.007 respectively).

Three classes of myogenic cells can be identified following injury: Pax7^+^/MyoD^+^ (expanding myoblasts), Pax7^−^/MyoD^+^ (committed to differentiation) and Pax7^+^/MyoD^−^ cells (self-renewing; Zammit et al, 2004). Following repeated injury, we observed a significant decrease in the number of self-renewing stem cells in Ku80 heterozygous and null muscles as compared with wildtype (Fig 3A and B). Furthermore, the numbers of expanding and committed cells increased in Ku80 heterozygous and null muscles suggesting impaired stem cell self-renewal (Fig 3B). We previously identified a population of interstitial muscle progenitors (PICs) that participate in post-natal growth (Mitchell et al, 2010). PICs are defined by their anatomical location in the interstitial space coupled with expression of PW1, whereas satellite cells are located under the basal lamina (Mauro, 1961) and can be identified by Pax7 (Seale et al, 2000), M-cadherin (Irintchev et al, 1994), and PW1 expression (Mitchell et al, 2010). To determine whether all muscle progenitors were affected in Ku80 null and heterozygous muscles, we examined 0- and 15-day-old muscles (Fig 3C–F). At birth, no significant differences were observed between null and wildtype muscles (Fig 3D–F). In contrast, we noted a significant decrease in the number of both satellite cells and PICs in 15 days Ku80 null muscles compared to wildtype (Fig 3D–F). No significant differences in the number of satellite cells and PICs were observed between wildtype and heterozygous muscles at all stages examined (Fig 3D–F).

### p53 activation and DNA damage in aging and Ku80 mutant muscle

**Figure 4:**
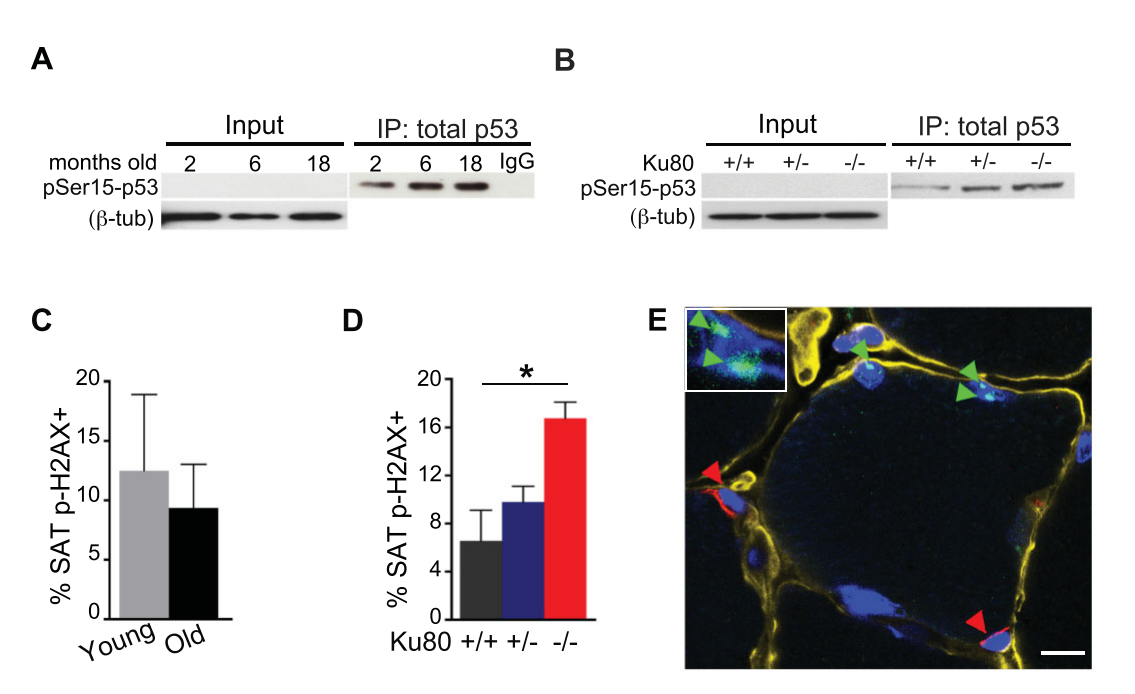
p53 activity increases with age and in response to loss of Ku80 Western analyses showing levels of activated p53 (phospho serine 15) in 2, 6 and 18-month-old Ku80^+/+^ skeletal muscle. p53 is only detectable by immunoprecipitation (input *vs*. IP).Western analyses showing elevated levels of activated p53 in 2-month-old Ku80^+/−^ and Ku80^−/−^ muscles compared to Ku80^+/+^. p53 is only detectable by immunoprecipitation (input *vs*. IP).Proportion of phospho-H2AX^+^ cells in satellite cells isolated from young (2 months old) and old (18 months old) skeletal muscle. No significant differences are observed.Proportion of phospho-H2AX^+^ cells in satellite cells isolated from 2-month-old Ku80^+/+^ (grey bars), Ku80^+/−^ (blue bars) and Ku80^−/−^ (red bars) muscles. Ku80^−/−^ muscle show increased percentage of phospho-H2AX^+^ satellite cells as compared to Ku80^+/+^ (*p = 0.047).Representative cross-section from 18-month-old muscle stained for M-cadherin (red), phospho-H2AX (green), Laminin (orange) and DAPI. A representative area is shown at higher resolution. Red arrows: M-cadherin^+^ satellite cells. Green arrows: foci formed by phospho-H2AX at DNA damage sites. Scale bar = 10 µm.

We next examined levels of activated p53 at 2, 6 and 18 months in wildtype hindlimb muscle and in 2-month-old Ku80 heterozygote and null muscles. We found that activated p53 levels increased with age in wildtype muscle (Fig 4A). Furthermore, Ku80 heterozygous and null muscles displayed high levels of activated p53 by 2 months of age comparable with 18 months wildtype muscle (Fig 4B). We next investigated levels of DNA damage in quiescent muscle stem cells in 2-month-old Ku80 wildtype, heterozygous and null as well as wildtype 18-month-old muscles using phospho-H2AX (Fig 4C–E). No differences were observed in the proportion of phospho-H2AX labelled satellite cells between young and old wildtype muscles (Fig 4C). We detected sparse labelling restricted to myonuclei in old muscles (Fig 4E). In contrast, Ku80 null muscle displayed a ∼2.5-fold increase in labelled satellite cells compared to Ku80 heterozygous and wildtype (Fig 4D). These data demonstrate that while loss of both Ku80 alleles leads to DNA damage in muscle stem cells, DNA damage does not accumulate during normal aging nor in response to the loss of a single Ku80 allele.

### Partial and complete loss of Ku80 alter myoblast proliferation and differentiation

**Figure 5:**
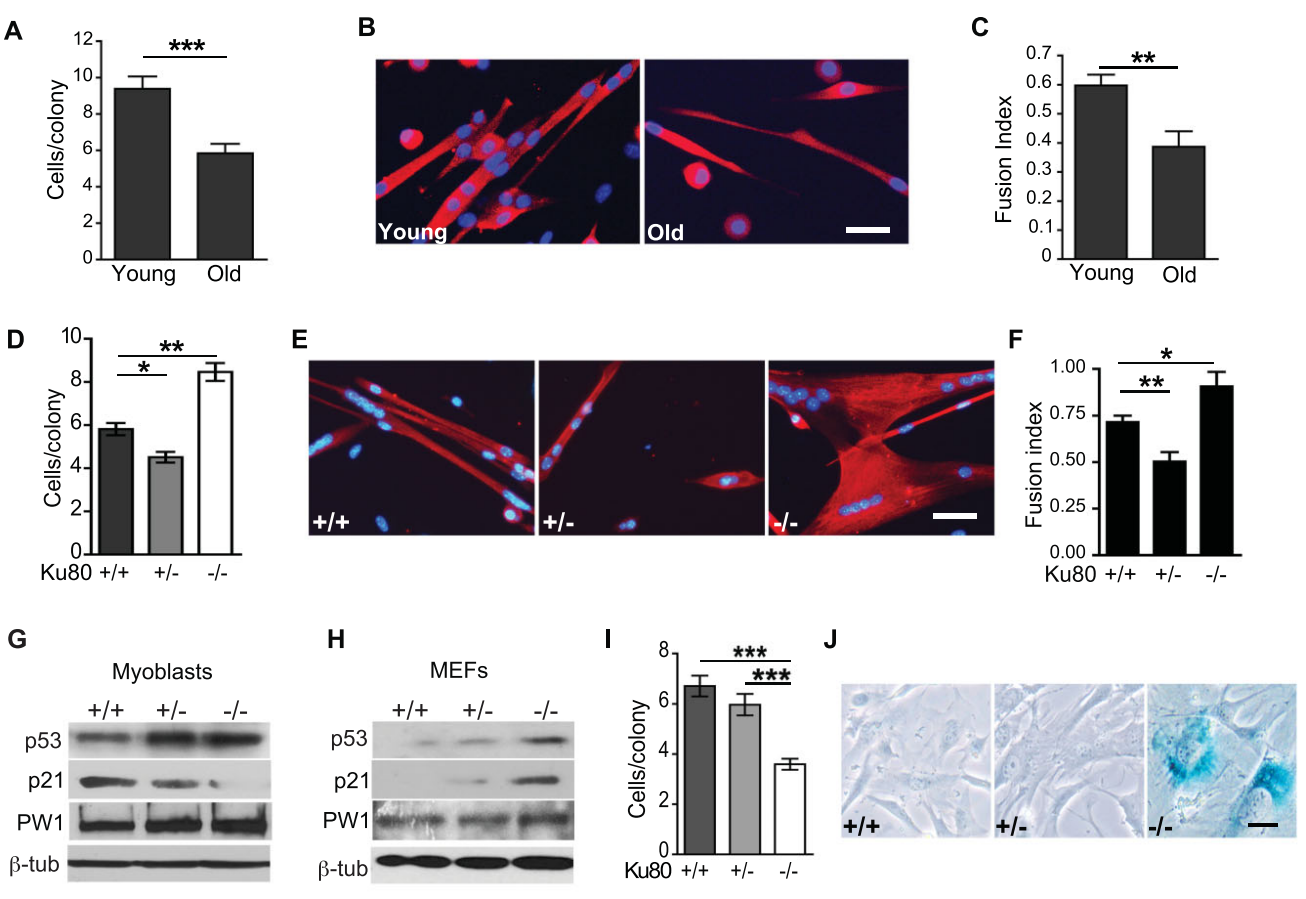
Myogenic stem cells and fibroblasts show different responses to complete and partial loss of Ku80 Histogram showing colony size generated by myoblasts purified from young (2 months old) and old (18 months old) Ku80^+/+^ muscles. Myoblast proliferative capacity decreases with age as determined by final colony size (***p < 0.0001).Young and old differentiated Ku80^+/+^ myoblasts immunostained for MHC (red) and DAPI (blue). Scale bar = 15 µm.Fusion index of Young and Old Ku80^+/+^ myoblasts stained as shown in (**B**). Old myoblasts show decreased myogenicity (**p = 0.004).Colony size generated from young Ku80^+/+^, Ku80^+/−^ and Ku80^−/−^ myoblasts. Ku80^+/−^ myoblast proliferation is impaired (*p = 0.05) while Ku80^−/−^ myoblasts exhibit an increased proliferative capacity compared to Ku80^+/+^ (**p = 0.008).Differentiated Ku80^+/+^, Ku80^+/−^ and Ku80^−/−^ myoblasts, immunostained for MHC (red) and DAPI (blue). Ku80^+/−^ myoblasts differentiated poorly whereas Ku80^−/−^ myoblasts formed very large myotubes. Scale bar = 15 µm.Fusion index of young Ku80^+/+^, Ku80^+/−^ and Ku80^−/−^ myoblasts stained as shown in (**E**) (*p = 0.014 and **p = 0.022).Western analyses showing activated p53, p21 and PW1 levels in Ku80^+/+^, Ku80^+/−^ and Ku80^−/−^ myoblast nuclei. Additional quantification and transfer verification is shown in Supporting Information Fig S3A and B.Western analyses showing active p53, p21 and PW1 in Ku80^+/+^, Ku80^+/−^ and Ku80^−/−^ MEF nuclei. Additional quantification is shown in Supporting Information Fig S3A and B.Histogram showing colony sizes generated from Ku80^+/+^, Ku80^+/−^ and Ku80^−/−^ MEFs. Ku80^−/−^ MEFs form smaller colonies as compared to Ku80^+/+^ and Ku80^+/−^ MEFs (***p < 0.0001).Endogenous β-galactosidase activity is detected in Ku80^−/−^ MEFs indicating cellular senescence. No activity is detected in Ku80^+/+^ and Ku80^+/−^ MEFs. Scale bar = 10 µm.

We examined the colony size generated by myoblasts purified from 2-month-old Ku80 wildtype, heterozygous and null muscles and compared it with myoblasts generated from 18-month-old wildtype muscles. We found that colony sizes decreased with age (Fig 5A). When challenged to differentiate, all myoblasts formed multinucleated myotubes expressing MHC, however, myoblasts from ‘old’ wildtype muscle formed smaller myotubes containing less nuclei as compared to myoblasts from ‘young’ muscle (Fig 5B and C). Myoblasts derived from ‘young’ Ku80 heterozygous muscle formed smaller colonies compared to wildtype (Fig 5D). Surprisingly, Ku80 null myoblasts generated larger colonies (Fig 5D) and displayed a more robust differentiation consisting of large myotubes, whereas heterozygote myoblasts formed small myotubes with fewer nuclei as compared to wildtype and null myoblasts (Fig 5D–F). The fusion index of Ku80 null myoblasts showed a significant increase compared to both wildtype and heterozygous myoblasts (Fig 5F). These data demonstrate that Ku80 null myoblasts have a higher proliferative differentiation capacity whereas heterozygote myoblasts more closely resemble ‘old’ myoblasts that have a poor fusion index and low-proliferative capacity. Consistent with the observed increase in levels of activated p53 in Ku80 heterozygous and null muscles (Fig 4B), we found that activated p53 levels were increased in cultured Ku80 heterozygous and null myoblasts compared to wildtype (Fig 5G and Supporting Information Fig S3).

The cell cycle gene p21 is a primary p53 target activated in response to stress and DNA damage (Vousden, 2000). Surprisingly, p21 levels were moderately decreased in Ku80 heterozygous myoblasts and almost completely abrogated in Ku80 null myoblasts despite increased levels of activated p53 (Fig 5G and Supporting Information Fig S3). PW1 is another p53 target involved in cell stress (Coletti et al, 2002; Relaix et al, 2000) and, as expected, PW1 levels are increased in Ku80 heterozygous and null myoblasts (Fig 5G). Whereas p53 activation leads to cell cycle exit as well as cell death (Vousden, 2000), we did not observe cell senescence/death in the Ku80 null and heterozygous myoblasts. These observations demonstrate that Ku80 null myoblasts behave differently than Ku80 null mouse embryonic fibroblasts (MEFs), which proliferate poorly and undergo precocious cellular senescence (Lim et al, 2000; Zhao et al, 2009). To exclude the possibility that our results were due to specific laboratory conditions, we derived MEFs from Ku80 wildtype, heterozygote and null mice. We observed that Ku80 null MEFs showed elevated levels of nuclear p53, p21 and PW1, as well as reduced proliferation coupled with an early onset of cellular senescence as demonstrated by the presence of flattened cells and staining for senescence-associated β-galactosidase activity (Dimri et al, 1995; Fig 5H–J and Supporting Information Fig S3). Taken together, these data reveal that myogenic stem cells and fibroblasts respond differently to the partial and complete loss of Ku80.

### p53 regulates skeletal post-natal muscle maturation and aging

**Figure 6:**
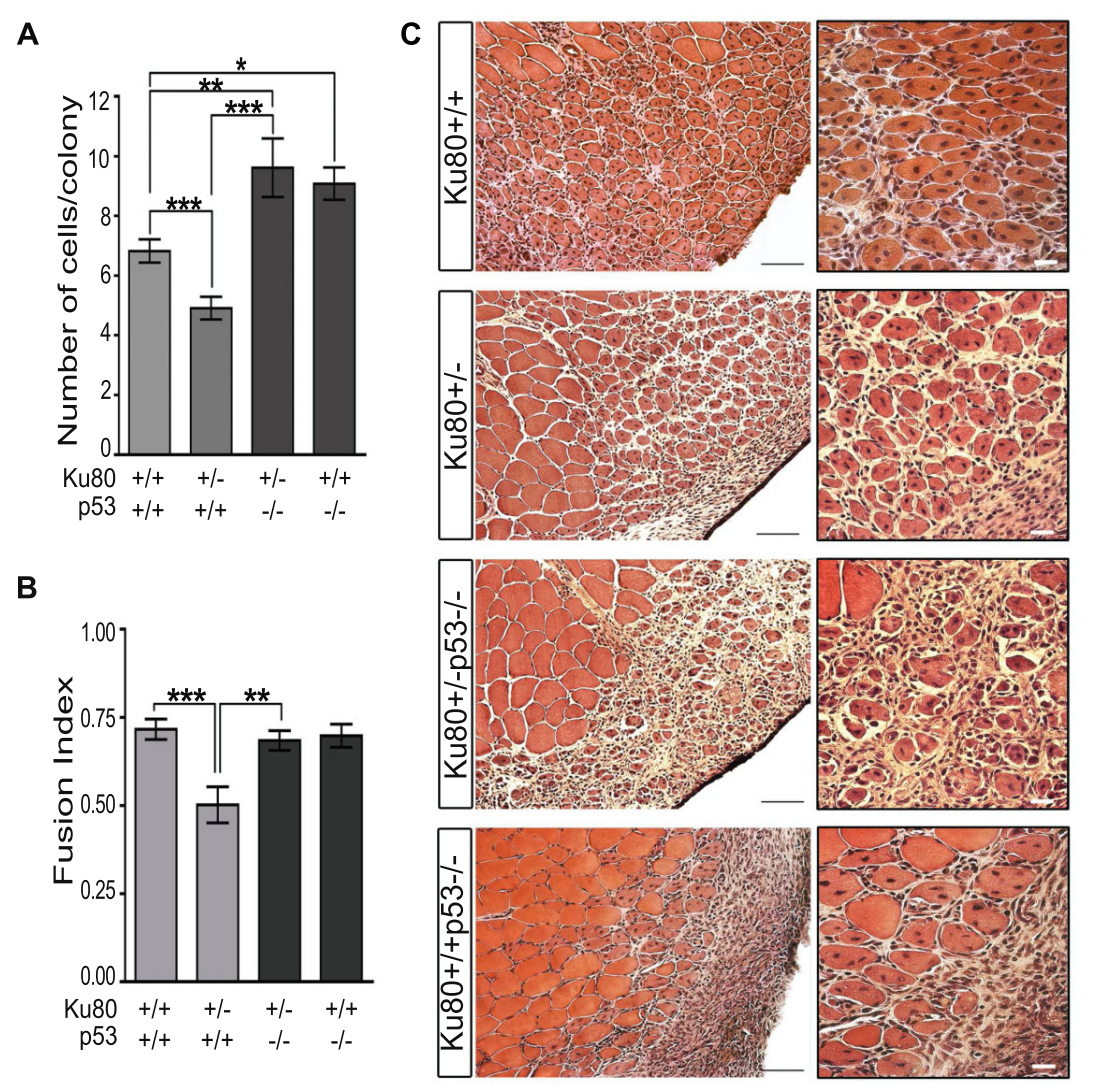
p53 deletion partially rescues Ku80^+/−^ and Ku80^−/−^ muscles **A-B.** Myoblast colony (**A**) and fusion index (**B**) from Ku80^+/+^ and Ku80^+/−^ p53 WT and from Ku80^+/+^, and Ku80^+/−^ on a p53 null background. Loss of p53 restores Ku80^+/−^ myoblast proliferation (*p = 0.011, **p = 0.002, ***p < 0.0001) (**A**), and fusion (**B**) as compared to Ku80^+/−^ and Ku80^+/+^ (**p = 0.007, ***p = 0.0004).**C.** *TA* cross-sections stained with hematoxylin/eosin 7 days following injury. Scale bar = 100 µm. A representative area of each cross section is shown at higher resolution. Scale bar = 20 µm. Deletion of p53 does not rescue Ku80^+/−^ muscle regeneration.

We generated Ku80 heterozygous mice on a p53 null background to assay the role of p53 in this context. We excluded Ku80:p53 double homozygous mutants as they rarely live beyond several weeks after birth [(Lim et al, 2000) and our unpublished observations]. Muscles from 2-month-old Ku80 heterozygous mice exhibited a reduced proportion of mixed (I/IIa) fibres compared to Ku80 wildtype muscle suggesting that Ku80 heterozygous muscles undergo an accelerated maturation or aging as compared to wildtype muscle at the same age (Supporting Information Fig S4). Loss of p53 rescued the wildtype fibre profile in Ku80 heterozygous muscles (Supporting Information Fig S4). Loss of p53 alone (Ku80^+/+^) did not significantly change fibre proportions as compared to wildtype muscle (Supporting Information Fig S4). Furthermore, loss of p53 rescued the proliferative and fusion capacity of Ku80 heterozygous myoblasts compared to Ku80 wildtype myoblasts; however, we noted that loss of p53 alone also results in an increased proliferative capacity (Fig 6A and B). We next tested whether p53 participates with Ku80 in muscle regeneration. As shown in Fig 2A–C, muscle regeneration is impaired in Ku80 heterozygous muscle. In contrast to the rescue of muscle fibre type and myoblast proliferation and differentiation, we found that muscle regeneration was further abrogated in Ku80 hetero:p53 null muscle as compared to the Ku80 heterozygous muscle. In particular, we noted larger interstitial spaces and more infiltrating cells (Fig 6C). Thus, while loss of p53 restores normal ‘young’ muscle fibre type profiles, it does not rescue the wildtype muscle regenerative capacity in Ku80 heterozygous mice.

### Partial or complete loss of Ku80 leads to telomere shortening in muscle stem cells

**Figure 7:**
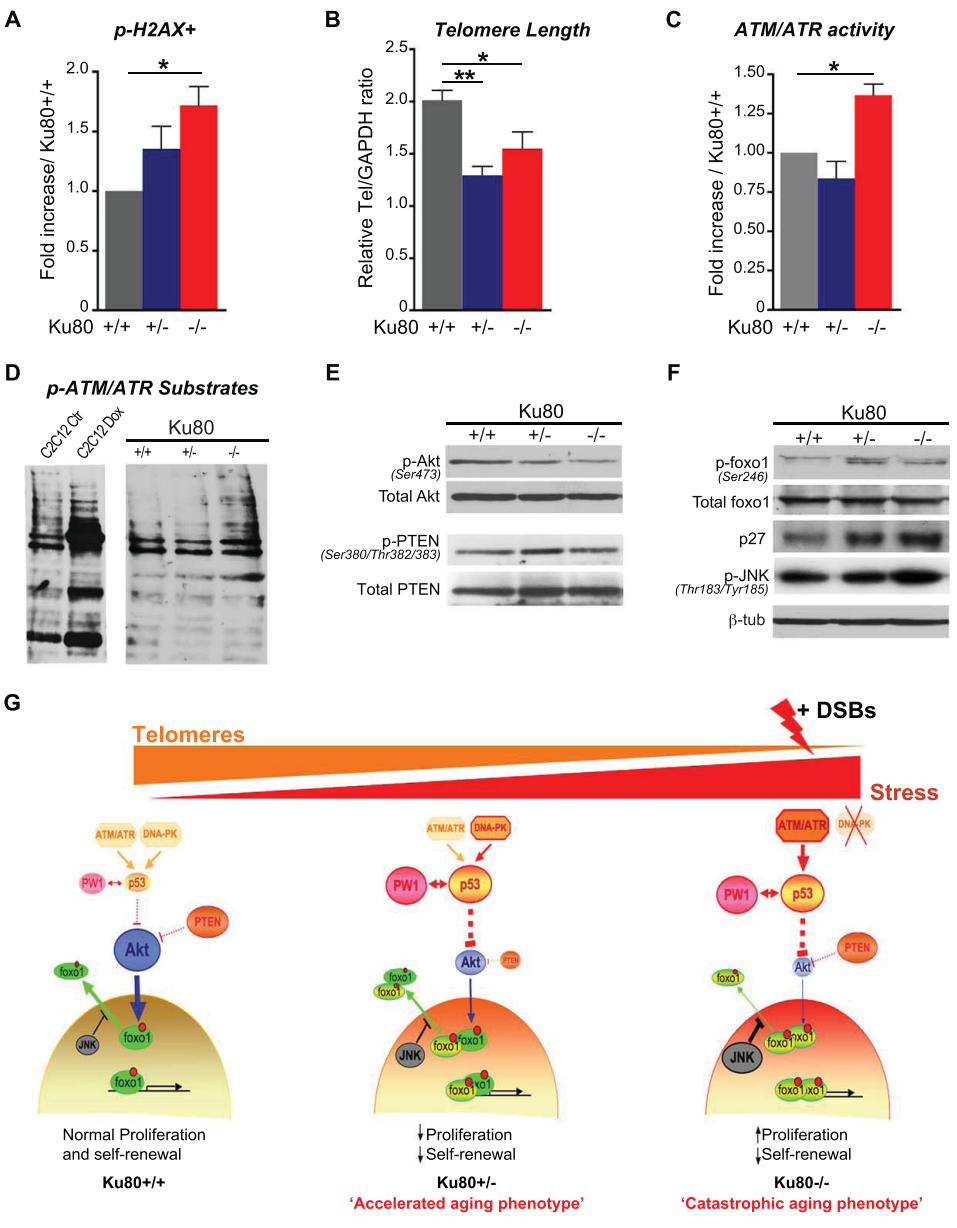
Loss of Ku80 leads to telomere attrition and deregulates the IGF-I/Akt pathway Histogram showing the proportional fold increase of phospho-H2AX^+^ myoblasts in Ku80^+/−^ and Ku80^−/−^ compared to Ku80^+/+^. Phospho-H2AX^+^ myoblasts are increased in Ku80^−/−^ compared to Ku80^+/+^ (*p = 0.03).Telomere lengths are shortened in Ku80^+/−^ and Ku80^−/−^ myoblasts as compared to wildtype evaluated by real-time PCR (*p = 0.046, **p = 0.009).Histogram of ATM/ATR activity in Ku80^+/−^ and Ku80^−/−^ myoblasts compared to Ku80^+/+^, quantified by flow cytometry. Ku80^−/−^ myoblasts exhibit increased ATM/ATR activity compared to Ku80^+/+^ (*p = 0.04).Western analyses showing phospho-(Ser/Thr) ATM/ATR substrates in proliferating C2C12 untreated or treated with Doxorubicin as a control, and Ku80^+/+^, Ku80^+/−^ and Ku80^−/−^ myoblasts. Ku80^−/−^ myoblasts display increased levels of phospho-ATM/ATR substrates compared to Ku80^+/+^ and Ku80^+/−^.Western analyses showing phospho-Akt(Ser473), total Akt, phospho-PTEN (Ser380/Thr382/383) and total PTEN in Ku80^+/+^, Ku80^+/−^ and Ku80^−/−^ myoblasts. Changes in levels are shown in Supporting Information Fig S5.Western analyses showing nuclear levels of phospho-foxo1(Ser246), total foxo1, p27 and phospho-JNK(Thr183/Tyr185) in Ku80^+/+^, Ku80^+/−^ and Ku80^−/−^ myoblasts.Proposed model for muscle stem cell dysfunction in Ku80^+/+^, Ku80^+/−^ and Ku80^−/−^ muscles. Dashed lines represent indirect effects. Thickness and colours of lines are proportional to kinase activities (see Discussion for description).

Our results revealed no significant accumulation of DNA damage in muscle stem cells of Ku80 heterozygous mice, however, muscle progenitors rarely cycle in undamaged muscle. We therefore compared DNA damage levels using phospho-H2AX labelling in myoblasts grown under proliferative conditions. Consistent with our *in vivo* observations, we observed a ∼1.7-fold increase in the proportion of Ku80 null phospho-H2AX-positive myoblasts compared to wildtype. In contrast, Ku80 heterozygous myoblasts showed a weak increase in labelling (Fig 7A). As Ku80 heterozygous myoblasts display elevated levels of activated p53, we reasoned that they respond to another source of cell stress. Telomere attrition has been proposed as a main source of cell stress associated with age (Flores et al, 2005; Sacco et al, 2010). Since Ku80 is involved in maintenance of telomere stability (D'Adda di Fagagna et al, 2001), we investigated telomere lengths in Ku80 wildtype, heterozygous and null cultured myoblasts. We found a significant decrease in telomere length in Ku80 heterozygous and null myoblasts as compared with wildtype (Fig 7B). We next investigated ataxia telangiectasia mutated (ATM) and Rad-3 related (ATR), which are the two principal effector kinases in response to telomere shortening (Denchi & de Lange, 2007). Surprisingly, we observed an increase of ATM/ATR activity only in Ku80 null myoblasts compared to heterozygous and wildtype (Fig 7C and D) revealing a unique response in the heterozygous progenitors in which this activity is blocked.

### IGF-1/Akt pathway is impaired Ku80 heterozygous and null muscle stem cells

Since loss of p53 does not completely rescue Ku80 heterozygous muscle, we investigated the IGF-1/Akt pathway, which is also inhibited by p53 (Feng et al, 2007). We observed a decrease in the proportion of the phosphorylated Akt (active) in Ku80 heterozygous and null myoblasts compared to wildtype (Fig 7E and Supporting Information Fig S5). Furthermore, PTEN activity was decreased in Ku80 heterozygous as compared to wildtype and null myoblasts (Fig 7E and Supporting Information Fig S5). Since phosphorylated PTEN is inactive and inhibits the Akt pathway, we propose that Ku80 heterozygous progenitors develop a compensatory mechanism to inhibit Akt-mediated inhibition. Since Foxo1 is a downstream effector of Akt in muscle and modulates cell cycle genes (Bois & Grosveld, 2003; Kamei et al, 2004; Machida et al, 2003), we investigated foxo1 levels and its target gene, p27, an inhibitor of the cell cycle, in primary muscle cells (Machida et al, 2003). Interestingly, we observed nuclear accumulation of two distinct isoforms of foxo1 that are phosphorylated by Akt (Fig 7F). Consistent with nuclear accumulation of foxo1, we observed increased levels of p27 in Ku80 heterozygous and mutant myoblasts (Fig 7F). As phosphorylation of foxo1 by Akt is expected to lead to foxo1 exclusion from the nucleus, we investigated levels of phosphorylated JNK, which can block Akt activity in response to stress (Sunayama et al, 2005). Consistent with nuclear accumulation of phospho-foxo1, we observed high levels of pJNK in myoblast nuclei (Fig 7F). Furthermore, pJNK is increased in Ku80 heterozygous and mutant myoblasts as compared to wildtype. We performed the same analyses in MEFs (Supporting Information Fig S5) and as expected, Ku80 wildtype MEF nuclei do not display detectable levels of phosphorylated foxo1, in contrast to Ku80 mutant MEF nuclei. Taken together, these data reveal that stress signalling follows distinct pathways in myogenic precursors compared to fibroblasts.

## DISCUSSION

The decline in muscle function and mass in the elderly is a major health issue, thus defining appropriate models with short lag times is important for therapy development. Skeletal muscle aging phenotypes occur late in life in mice (∼18 months), consequently experimental models are cumbersome and costly. Murine models for accelerated aging, including Ku80 mutant mice, show a severe phenotype at birth and poor post-natal growth and survival. While these mutations lend insight into progeric diseases, the applicability of these models to understanding normal aging in muscle is unclear. Consistent with the severe progeric phenotype reported in other tissues, we observed premature aging in Ku80 mutant muscle in young mice (<2 months) normally found in old mice (∼18 months). Ku80 heterozygous mice had been described as normal. However, in this study, we showed that skeletal muscle displays early onset aging. Specifically, we observed that heterozygote muscle showed poor regenerative capacity following a single injury coupled with poor stem cell self renewal as well as a premature shift to an ‘aged’ fibre type. The Ku80 heterozygous mouse is an attractive model for aging research as it better reflects the situation found in physiological aging. The Ku80 heterozygous mouse displays normal post-natal growth followed later by early onset aging in muscle. Whether this phenotype is limited to skeletal muscle remains to be investigated.

Several studies report a decline in satellite cell number with age in rodents and humans (Brack et al, 2005; Renault et al, 2002), whereas others contradict these findings (Conboy et al, 2003, 2005; Dreyer et al, 2006; Roth et al, 2000). Results presented in this study may address these discrepancies. We observed a marked depletion of two key muscle progenitor populations, satellite cells and PICs, by 15 days after birth in Ku80 null muscles. In contrast, the decrease was not statistically significant in the Ku80 heterozygous muscle, however, muscle progenitors exhibited decreased self-renewal coupled with poor myogenicity following injury. Thus, in the Ku80 heterozygous muscle, the predicted decrease in progenitor cells would be less pronounced than in null muscle and would depend upon the degree of additional stress incurred such as injury or genotoxic stress. In contrast, the Ku80 null muscle showed poor post-natal growth and reduced cellularity although fibre number was not reduced. Paradoxically, Ku80 null myoblasts showed an increased proliferative capacity and a rapid and efficient regenerative response *in vivo*. Following two consecutive injuries, regenerative capacity was lost coupled with lower progenitor self-renewal. We conclude that the aging response in both the null and heterozygous muscle is in part due to changes in stem cell competence.

**The paper explained**

PROBLEM:

Age-related diseases such as sarcopenia pose serious socio-medical issues as the aging population increases worldwide. Research using animal models either requires long study periods to allow for physiological aging or genetic models that display accelerated aging (progeric models). Identifying tractable models is critical to the design of effective therapeutic targets to treat sarcopenia and other age-related diseases.

RESULTS:

The Ku80 null mouse undergoes accelerated aging. Analysis of young Ku80 null mice revealed a fibre type profile consistent with accelerated aging. Surprisingly, Ku80 heterozygous mice also showed signs of accelerated aging. However, unlike the knockout mice, Ku80 heterozygous mice grow to a normal adult size. Nonetheless, their skeletal muscle has poor regenerative capacity coupled with low stem cell self-renewal. The Ku80 heterozygous mouse stem cells undergo premature telomere shortening confirming a role for Ku80 in telomere maintenance. Telomere shortening leads to a chronic cell stress response including the activation of p53 that is also increased in aged wildtype mice.

IMPACT:

These data provide a tractable model for the study of physiological aging. In addition, these results point to a key role for resident stem cells in the maintenance of skeletal muscle tissue integrity with age.

Growing evidence indicates that p53 is a regulator of aging. Additional p53 alleles increase tumour resistance and lifespan (Matheu et al, 2007) whereas chronically elevated levels of activated p53 lead to early onset aging (Cao et al, 2003; Maier et al, 2004; Tyner et al, 2002; Varela et al, 2005; Vogel et al, 1999). p53 is implicated in stem cell regulation (Cicalese et al, 2009; Hong et al, 2009; Kawamura et al, 2009; Li et al, 2009; Marion et al, 2009; TeKippe et al, 2003). We and others have demonstrated a role for p53 in the regulation of skeletal muscle stem cells, muscle homeostasis, and aging (Chung & Ng, 2006; Machida & Booth, 2004; Schwarzkopf et al, 2006; Siu & Alway, 2005). We show here that activated p53 levels increase with age and in the Ku80 heterozygous and null muscle indicating the presence of age-related stress signalling. Telomere shortening in stem cells is proposed to lead to tissue aging (Blasco et al, 1997; Sacco et al, 2010). Of particular interest is the murine model for Duchenne dystrophy (mdx) that does not display the severe myopathy seen in boys unless it is crossed to a mouse deficient for telomerase (Sacco et al, 2010). Telomerase deficiencies may not be evident unless stem cells are frequently recruited as in the case of mdx. Under proliferative conditions *in vitro*, we observed a decrease of telomere length in Ku80 heterozygous and null myoblasts as compared to Ku80 wildtype. Despite the long telomere lengths in mice as compared with humans (Kipling & Cooke, 1990), our data and that of others suggests a key role for telomere maintenance in stem cells regulation (Ferron et al, 2009; Sacco et al, 2010). We propose that in ‘young’ muscle, p53 activity remains at basal levels providing a balance between stem cell self-renewal and differentiation. With age, p53 activity increases due to telomere shortening. This model predicts that if telomere length is not maintained, aging is accelerated, but post-natal body growth is normal. Indeed, Ku80 heterozygous mice do not display an overt phenotype, however, when skeletal muscle is examined in detail, we find clear features of premature aging. Our data provide a model for muscle stem cell behaviour during aging (Fig 7G). Partial loss of Ku80 leads to telomere shortening in muscle stem cells. Progenitors also develop a protective mechanism to limit ATM/ATR activation and consequent DNA damage responses. This leads to a moderate but accelerated aging phenotype by 2 months of age as opposed to 18 months in the wildtype. Complete loss of Ku80 leads to telomere shortening coupled with an accumulation of DNA damage in which case ATM/ATR activity is strongly elevated with an end result of catastrophic accelerated aging. The interactions between p53 and the IGF1-Akt pathways provide a balance between positive signals that maintain or improve muscle function and negative signals that lead to muscle atrophy and a decline in regenerative competence through controlling cell cycle and cell fate in muscle progenitors.

## EXPERIMENTAL PROCEDURES

### Mice

Murine models: Ku80 and p53 lines (The Jackson Laboratory). Animal studies were carried out in adherence to French, European and NIH (USA) guidelines.

### Regeneration assays

Skeletal muscle regeneration was induced by focal freeze crush injury. Muscles were analysed 7 days after injury. For double regeneration experiments, a second injury was performed with a 15 days interval, and muscles were collected 7 days following the second injury.

### Histological and cells analyses

Muscles were snap frozen in liquid nitrogen-cooled isopentane as previously described (Nicolas et al, 2005). Cryosections (10 µm) were stained with haematoxylin and eosin. Collagen deposition was detected by Sirius Red staining (Lopez-De Leon & Rojkind, 1985), fat tissue was stained by Oil Red O and hematoxylin (Koopman et al, 2001). NADH-TR diaphorase staining was used as a measure of mitochondrial content and oxidative capacity of fibres as previously described (Zhang et al, 2010). MHC isoforms and fibres size were measured from cryosections obtained from the mid-belly of Soleus stained with MHC2x/6H1, MHC2b/BFF3, MHC1/BAD5 and MHC2a/SC71 antibodies (Hybridoma bank) as previously described (Trollet et al, 2010). Images were captured on a Zeiss AxioImagerZ1 microscope, and morphometric analysis was performed using MetaMorph7.5 (Molecular Devices). Entire sections containing 700–1000 fibres were analysed from 3 to 4 animals per group. Distributions of fibre diameter were compared using Kolmogorov–Smirnov Test *p < 0.05. Sections and cultured cells were stained with antibodies against PW1, Pax7 (Developmental Studies Hybridoma Bank), laminin (Sigma), Mcadherin (NanoTools), Ki67 (BD Biosciences), MyoD (BD Biosciences and Santa Cruz), MF20 (Developmental Studies Hybridoma Bank), GFP (Biovalley) as previously described (Mitchell et al, 2010). Species specific secondary antibodies coupled to Alexa Fluor 488 (Molecular Probes), Cy3 or Cy5 (Jackson Immunoresearch) and nuclei were counterstained with DAPI (Sigma). For quantitative analysis, positive cells in at least 350 fibres from randomly chosen fields were counted from at least three animals per group. Values represent the mean ± SEM, and multiple group comparisons were performed using One-way ANOVA analysis followed by *post hoc* analysis.

### FACs analysis and primary culture

Hind-limb muscles were processed as previously described (Mitchell et al, 2010). Flow cytometry was performed on a FACS Aria (Becton Dickinson). Anti-phospho H2AX and anti-phospho-(Ser/Thr)ATM/ATR substrate antibodies (Cell signalling) were used for DNA damage and ATM/ATR activity. Purified cell populations were plated on gelatin-coated dishes at low density for clonal analysis. Cells were allowed to grow in 40% DMEM (GIBCO), 40% F-12 (GIBCO) containing 17% FBS (GIBCO), 1% v/v Penicillin–Streptomycin (GIBCO) and 2% Ultroser SF (Pall Bio Sepra), for 3 days. Myogenic differentiation was induced by shifting the medium to DMEM (GIBCO), containing 2% v/v horse serum (GIBCO) and 1% v/v Penicillin–Streptomycin (DM) for 3 days. Proliferative capacity was quantified by counting at least 100–150 colonies, from at least three independent experiments. Fusion index: the number of nuclei in MF20+ myotubes/total number of nuclei, of randomly chosen fields for minimum three independent experiments. Values represent mean ± SEM. Multiple group comparisons were performed using One-way ANOVA followed by *post hoc* analysis. MEFs were prepared from E13.5 embryos as previously described (Lengner et al, 2004). Endogenous β-galactosidase was assayed as previously described (Dimri et al, 1995).

### Telomere measurement

Telomere lengths were measured by RT-PCR amplification using 5 ng of genomic DNA and primers designed to hybridize to the telomere end repeats as previously described (Cawthon, 2002).

### Immunoprecipitation and Western analyses

Muscles were homogenized in a lysis buffer (150 mM NaCl, 50 mM Hepes pH7.6, 1% NP-40, 0.5% Sodium deoxycholate, 5 mM EDTA) supplemented with 1 mM PMSF, Complete (Roche), 20 mM NaF, 10 mM β-glycerophosphate, 5 mM Na-pyrophsphate, and 1 mM Na-orthovanadate. 200 µg of protein were immunoprecipitated for each sample. Lysates were incubated with 2 µg of anti-p53 Pab421 antibody (Calbiochem), for 2 h at 4°C, prior to protein G-sepharose (Sigma) addition. Proteins were eluted by boiling the samples 5 min in the presence of Laemmli buffer containing 10% β-mercaptoethanol.

For cultured cells, total proteins were prepared using RIPA lysis buffer and nuclear enriched extracts were prepared as previously described (Thornborrow & Manfredi, 1999). Equal amounts of protein were separated by electrophoresis (10% SDS-polyacrylamid) and transferred to a PVDF membrane in 20% methanol transfer buffer. Membranes were probed with antibodies to phospho-p53 Ser15, total and phospho-Akt Ser473, total and phospho-PTEN Ser380/Thr382/383, total and phospho-FKHR Ser246, p27, phospho-(Ser/Thr) ATM/ATR substrate and phospho-JNK Thr183/Tyr185 (Cell Signaling). Antibody binding was visualized with HRP-conjugated secondary antibodies (Jackson ImmunoResearch) and followed by enhanced chemiluminescence (Pierce). Quantification was done with ImageJ Software from at least three independent experiments.

## Supplementary material

Detailed facts of importance to specialist readers are published as ”Supporting Information”. Such documents are peer-reviewed, but not copy-edited or typeset. They are made available as submitted by the authors.
