# Supplementary material for: Loss of a single allele for Ku80 leads to progenitor dysfunction and accelerated aging in skeletal muscle
Source: EMBO Mol Med. 2012 Aug 23;4(9):910–23. doi: 10.1002/emmm.201101075 (PMC3491824; doi:10.1002/emmm.201101075)
Supplement: Supplementary file 1 [file emmm0004-0910-SD1.pdf]

EMBO
Molecular Medicine

Manuscript EMM-2011-01075

Loss of a single allele for Ku80 leads to progenitor dysfunction and accelerated aging in skeletal muscle

Nathalie Didier, Christophe Hourdé, Helge Amthor, Giovanna Marazzi and David Sassoon

Corresponding author: David Sassoon, UPMC Paris VI/ INSERM

Review timeline:

Submission date:	13 November 2011
Accepted:	26 June 2012

Transaction Report:

No Peer Review Process File is available with this article, as the authors have chosen not to make the review process public in this case.
