# Supplementary material for: Loss of a single allele for Ku80 leads to progenitor dysfunction and accelerated aging in skeletal muscle
Source: EMBO Mol Med. 2012 Aug 23;4(9):910–23. doi: 10.1002/emmm.201101075 (PMC3491824; doi:10.1002/emmm.201101075)
Supplement: Supplementary file 2 [file emmm0004-0910-SD2.pdf]

Supplementary Information :

Didier et al., Loss of a single allele for Ku80 leads to progenitor dysfunction and accelerated aging in skeletal muscle.

Contents :

- Figure S1. Changes in body weight, fiber number and cellularity in Ku80 heterozygous, mutant and old WT mice as compared to wildtype muscle.

- Figure S2. Increased fibrosis in serially injured Ku80 heterozygous and null muscle.

- Figure S3. Quantification and verification of p53 and p21 levels in Ku80 mutant, heterozygous and wildtype myoblasts and MEFs.

- Figure S4. Loss of p53 restores fiber type profiles in Ku80 heterozygous mice.

- Figure S5. Analyses of cell stress pathways in myoblasts and MEFs from Ku80 mutant, heterozygous and wildtype muscle progenitors.

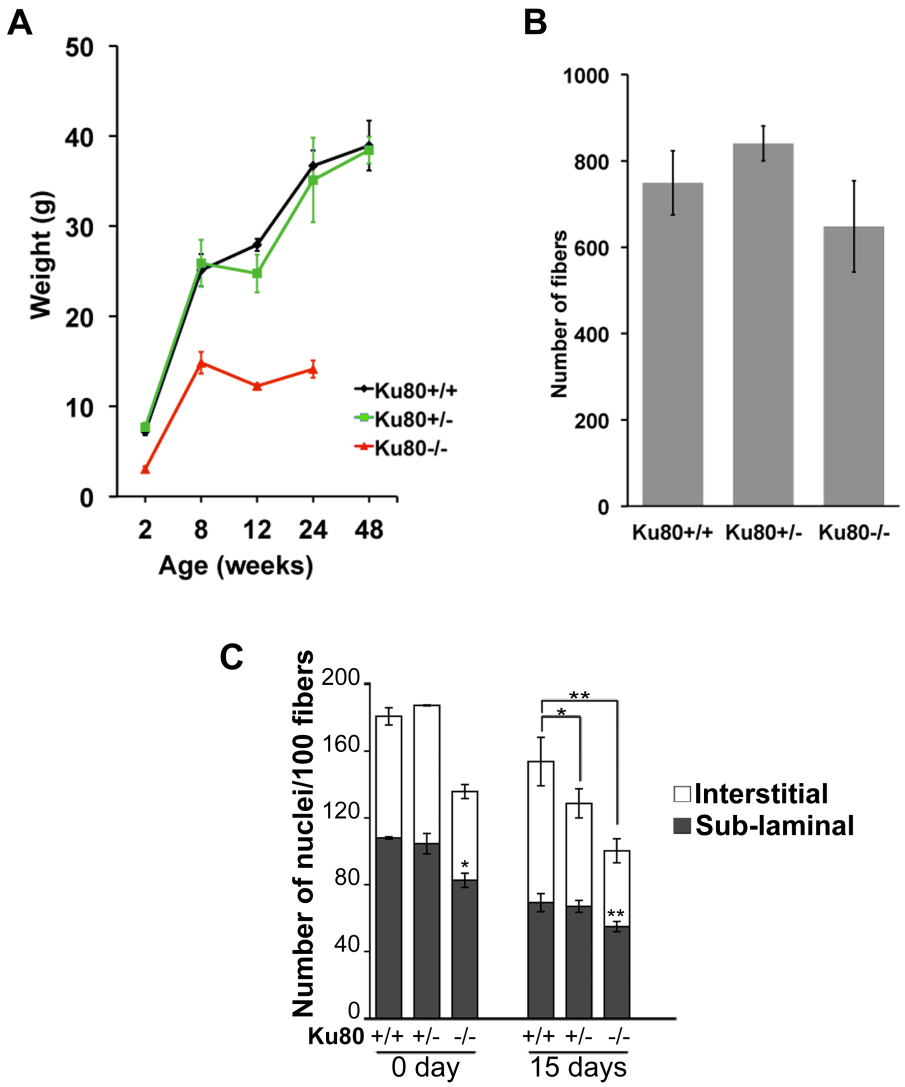

Figure S1

A) Body weight is significantly reduced in the Ku80 -/-. Changes in body weight with age in Ku80+/- (green), Ku80-/- (red) and Ku80+/+ (black) between 2 to 24 weeks. While the Ku80-/- displays a reduced postnatal growth as compared to Ku80+/+, we do not observe significant changes in Ku80+/- muscle weight.

B) Histogram showing the mean fiber number in Ku80+/+, Ku80+/-, and Ku80-/- 6 month old *Soleus*. No significant differences are observed.

C) Cellularity decreases post-natally and is further reduced in the Ku80 mutant and heterozygous muscle. Histogram showing the number of nuclei per 100 fibers from sections stained for laminin to delineate the muscle fibers, and DAPI to visualize nuclei. Ku80-/- muscles show a decrease number of sub-laminal nuclei as compared to Ku80+/+ both at 0 and 15 days (*p=0.02, **p=0.003). 15 days old Ku80+/- and Ku80-/- muscles exhibit a decreased number of interstitial nuclei as compared to Ku80+/+ muscles (*p=0.05 and **p=0.001).

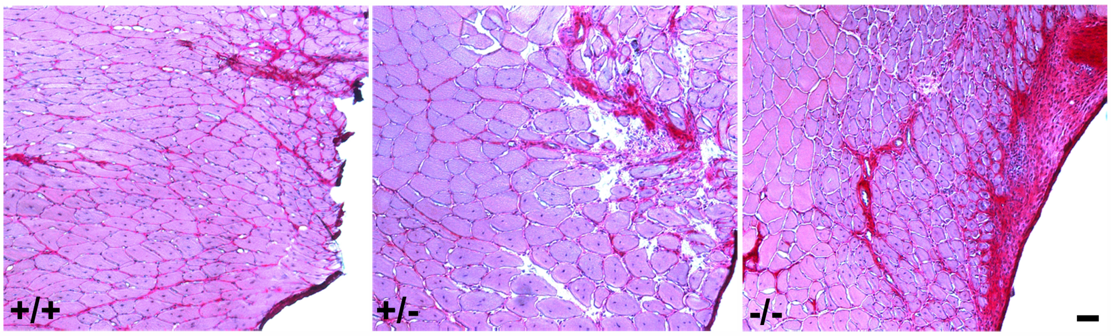

Figure S2. Increased fibrosis in serially injured Ku80 heterozygous and null muscle.

Representative photomicrographs of cross sections from adult *TA* stained with Sirius Red and hematoxylin following serial injury. We observed increased fibrosis in Ku80-/- and +/- muscle as compared to Ku80+/+ muscle. Scale bar=100μm.

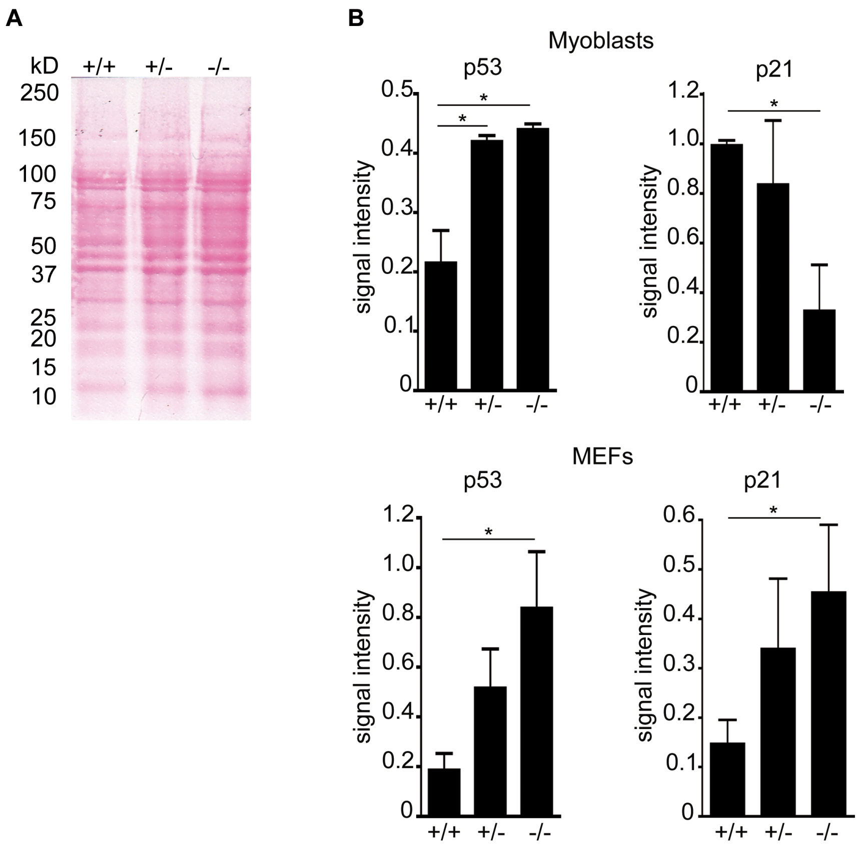

Figure S3. Quantification and verification of p53 and p21 in Ku80 +/+, +/-, and -/- myoblasts and MEFs.

A) Poinceau staining of transfer for Western blot shown in Figure 5G prior to immunodetection verifying equal presence of proteins in all samples.

B) (top left) Histogram showing quantification of multiple and independent Western analyses (n=3) for activated p53. Increased levels of p53 are seen in both Ku80 +/- (p=0.033) and Ku80-/- (p=0.028) as compared to wildtype.

(top right) Histogram showing quantification of multiple and independent Western analyses (n=3) for p21. Decreased levels of p21 are seen in Ku80-/- (p=0.0035) as compared to wildtype. No significant difference is seen between Ku80+/- and wildtype.

(lower left) Histogram showing quantification of multiple and independent Western analyses (n=3) for activated p53 in MEFs. Increased levels of p53 are seen in Ku80-/- (p=0.026) as compared to wildtype. The increase in p53 levels in Ku80+/- MEFs is not statistically significant although we note is trends upward.

(lower right) Histogram showing quantification of multiple and independent Western analyses (n=3) for p21 in MEFs. Ku80 -/- MEFs show a significant increase in p21 level (p=0.051) as compared to wildtype whereas the Ku80+/- show only a similar but not significant increase in p21 levels.

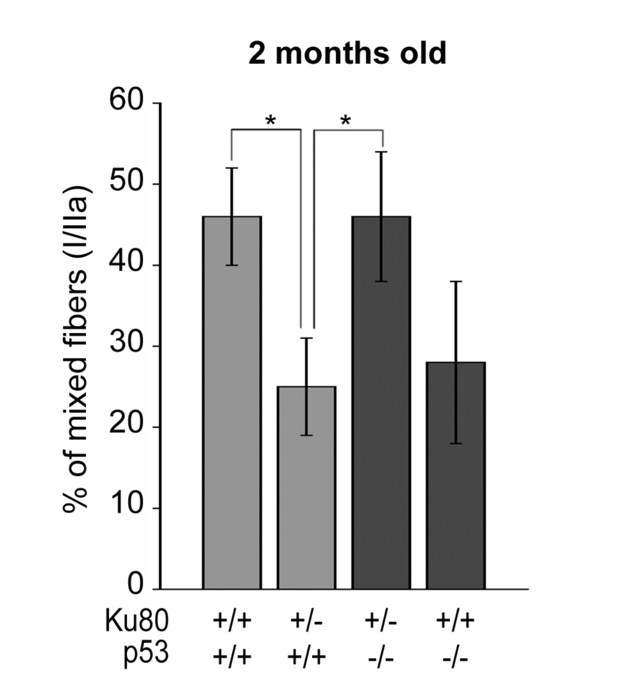

Figure S4. Loss of p53 restores fibertype profiles in Ku80 heterozygous mice. Histogram showing the proportion of mixed fiber population (I/IIa) in 2 months old *Soleus.* Ku80+/- *Soleus* display a reduced proportion of mixed fibers (I/IIa) as compared to Ku80+/+ (*p=0.014). Deletion of p53 restored the mixed fiber proportion to wildtype levels in Ku80+/- *Soleus* as compared to Ku80+/+ (*p=0.016). Results are shown as mean proportions ±SEM.

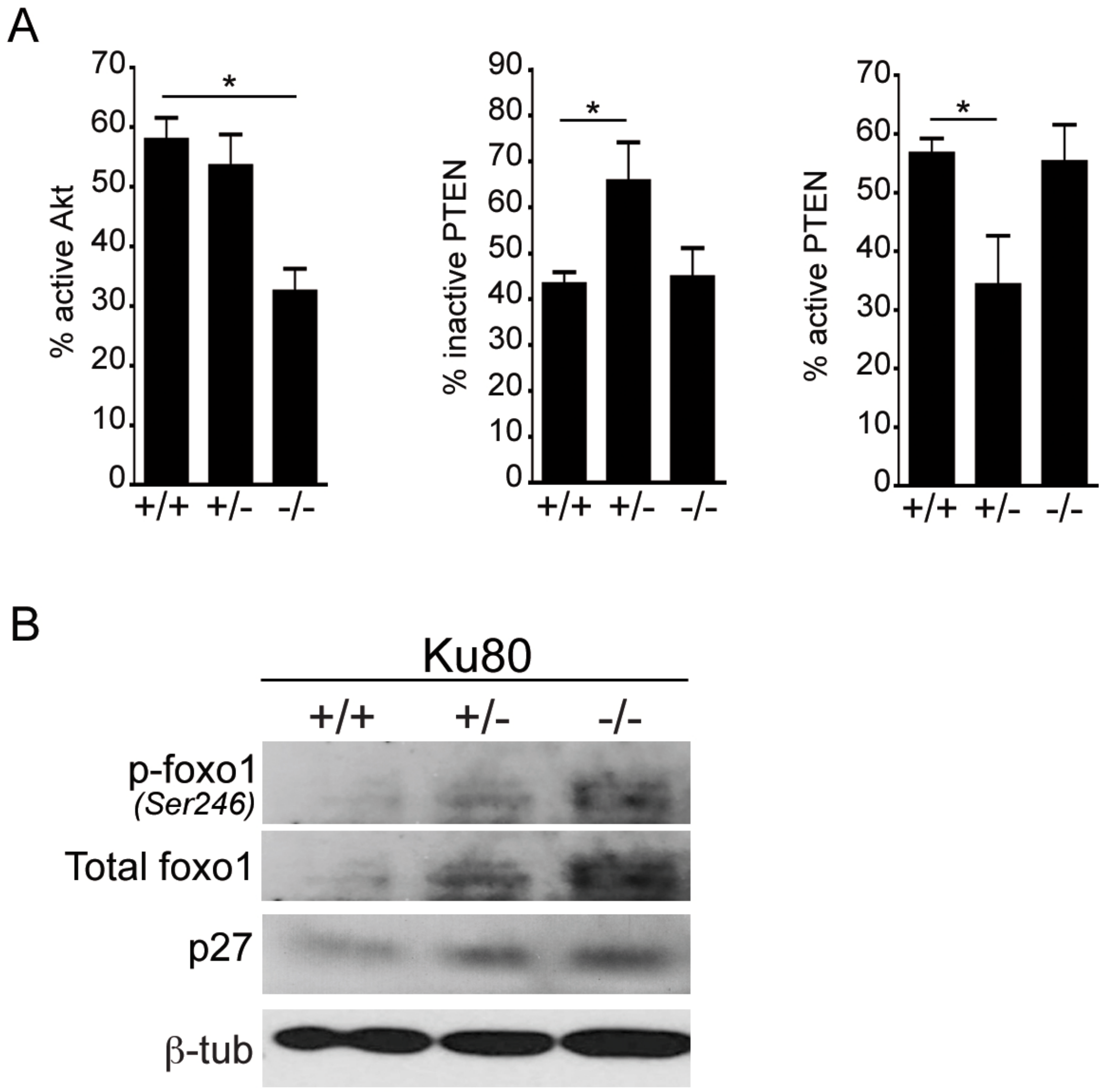

Figure S5. Analyses of cell stress pathways in myoblasts and MEFs from Ku80 mutant, heterozygous and wildtype muscle progenitors.

A) Histograms showing quantification of multiple and independent Western analyses (n=3) for active AKT (p=0.02), inactive (p=0.04) and active PTEN (p=04) in myoblasts.

B) Western analyses showing nuclear levels of phospho-foxo1(Ser246), total foxo1, and p27 in Ku80+/+, Ku80+/- and Ku80-/- MEFs.
